# The Complexity Measures Associated with the Fluctuations of the Entropy in Natural Time before the Deadly México M8.2 Earthquake on 7 September 2017

**DOI:** 10.3390/e20060477

**Published:** 2018-06-20

**Authors:** Alejandro Ramírez-Rojas, Elsa Leticia Flores-Márquez, Nicholas V. Sarlis, Panayiotis A. Varotsos

**Affiliations:** 1 Departamento de Ciencias Básicas, Universidad Autónoma Metropolitana Azcapotzalco, Av. San Pablo 180, Mexico City 02200, Mexico; 2 Instituto de Geofísica, Universidad Nacional Autonoma de Mexico, Mexico City 04510, Mexico; 3 Section of Solid State Physics and Solid Earth Physics Institute, Department of Physics, National and Kapodistrian University of Athens, Panepistimiopolis, 157 84 Zografos, Greece

**Keywords:** earthquakes, natural time analysis, complexity

## Abstract

We analyse seismicity during the 6-year period 2012–2017 in the new time domain termed natural time in the Chiapas region where the M8.2 earthquake occurred, Mexico’s largest earthquake in more than a century, in order to study the complexity measures associated with fluctuations of entropy as well as with entropy change under time reversal. We find that almost three months before the M8.2 earthquake, i.e., on 14 June 2017, the complexity measure associated with the fluctuations of entropy change under time reversal shows an abrupt increase, which, however, does not hold for the complexity measure associated with the fluctuations of entropy in forward time. On the same date, the entropy change under time reversal has been previously found to exhibit a minimum [Physica A 506, 625–634 (2018)]; we thus find here that this minimum is also accompanied by increased fluctuations of the entropy change under time reversal. In addition, we find a simultaneous increase of the Tsallis entropic index *q*.

## 1. Introduction

A widespread opinion is that the two recent deadly Mexico earthquakes, i.e., M8.2 on 7 September 2017 and M7.1 on 19 September 2017, in Chiapas and Morelos regions, respectively, had an unusual cause. In particular, although most big Mexican earthquakes occur along the interface between the subducting Cocos plate and the North American plate, in the case of the M8.2 earthquake that struck the Chiapas state, the earthquake occurred within the Cocos plate itself. Thus, since some seismologists say that this type of faulting should not produce such large earthquakes [1], they characterize it as an “extremely strange” event [2]. As for the M7.1 earthquake, which struck central Mexico on 19 September, it occurred at 57 km depth [3] also within the Cocos plate near the northern limit of the Mexican flat-slab [4,5], where it begins to plunge beneath the North American plate. In short, the two quakes happened at two different spots within the Cocos tectonic plate and surprised seismologists [6].

In a previous paper [7], upon considering the analysis of seismicity in the new time domain termed natural time, we showed that the occurrence of the M8.2 earthquake, Mexico’s largest earthquake in more than a century, should not be considered unexpected. Specifically, this analysis led to the result that in the Chiapas region, where the M8.2 earthquake occurred, the probability for the occurrence of an extreme event was the highest compared to other regions in Mexico. Furthermore, in this region, the same analysis revealed that the entropy change Δ*S* under time reversal exhibited a pronounced minimum on 14 June 2017, i.e., almost 3 months before the occurrence of the M8.2 earthquake, which pointed to the conclusion that an extreme event was likely to take place there in view of the following: Upon considering the Olami-Feder-Christensen (OFC) model for earthquakes [8], which is probably [9] the most studied non-conservative (supposedly), self-organized criticality (SOC) model (see also [10]), we found that the value of the entropy change under time reversal shows a clear minimum [11,12] before a large avalanche, which corresponds to a large earthquake.

The main purpose of this paper is to investigate the complexity measures associated with the fluctuations of either the entropy defined in natural time and/or the entropy change under time reversal of the seismicity in the Chiapas region from 01 January 2012 until 20 October 2017. In addition, we study whether, beyond the above mentioned precursory Δ*S* minimum reported in [7], there exist also similar changes in the complexity measures investigated here. These measures have been found to be useful when the system approaches the critical point (dynamic phase transition) as, for example, is the case in an impending sudden cardiac death risk ([13,14], see also Section 9.4.1 of Ref. [11]).

## 2. Natural Time Analysis. The Entropy Defined in Natural Time and the Associated Complexity Measures

For a time series of *N* events, we define an index for the occurrence of the *k*-th event by *χ_k_ = k/N*, which we term natural time *χ*. In this analysis [11,15], we ignore the time intervals between consecutive events, but preserve their order and energy *Q_k_*. We then study the pairs (*χ_k_*, *Q_k_*) or the pairs (*χ_k_*, *p_k_*) where

(1) $$p_{k}=\frac{Q_{k}}{\sum_{k=1}^{N}Q_{k}}$$

is the normalized energy for the *k*-th event. The entropy *S* in natural time is defined [13,16,17] by

(2) S=〈χlnχ〉−〈χ〉ln〈χ〉

where the brackets <…> ≡ ∑(…)*p_k_* denote averages with respect to the distribution *p_k_*, i.e., <*f*(*χ*)> ≡ ∑*f*(*χ_k_*)*p_k_*. The entropy *S* is a dynamic entropy that exhibits [18] concavity, positivity, and Lesche stability [19,20].

Upon considering time reversal $\hat{T}$, i.e., $\hat{T}$*p_k_* = *p_N−k+_*_1_, the value of *S* changes to a value *S*_−_:

(3) $$S\_=\sum_{k=1}^{N}\frac{k}{N}\ln\left(\frac{k}{N}\right)p_{N-k+1}-\left(\sum_{k=1}^{N}\frac{k}{N}p_{N-k+1}\right)ln\left(\sum_{l=1}^{N}\frac{l}{N}p_{N-l+1}\right)$$

The physical meaning of the change of entropy ∆*S* ≡ *S − S_−_* in natural time under time reversal has been discussed in References. [11,21,22].

Using a moving window of length *i* (number of consecutive events) sliding through the time series of *L* consecutive events, the entropy in natural time has been determined for each position *j =* 1, 2, …, *L − i* of the sliding window. Thus, a time series of *S_i_* has been constructed [13] whose standard deviation is designated by *δS_i_*. The study of the effect of the change of scale *i* on *δS_i_* is made [14] by means of the complexity measure

(4) $$\lambda_{i}=\;\frac{\delta S_{i}}{\delta S_{100}}$$

where the denominator δ*S*_100_ is arbitrarily selected to stand for the *δS* value of a short scale, i.e., 100 events, while the numerator corresponds to a longer scale, e.g., *i* = 10^3^ events. If instead of *δS_i_* the standard deviation *σ*(Δ*S_i_*) of the time series of Δ*S_i_* ≡ *S_i_ −* (*S_−_*)*_i_* is used, we define [11,23] the complexity measure Λ*_i_*,

(5) $$\Lambda_{i}=\;\frac{\sigma \left(\Delta S_{i}\right)}{\sigma \left(\Delta S_{100}\right)},$$

when a moving window of *i* consecutive events is sliding through the time series and the denominator *σ*(Δ*S*_100_) is arbitrarily selected to correspond to the standard deviation *σ*(Δ*S*_100_) of the time series of Δ*S_i_* of *i* = 100 events. In other words, this complexity measure quantifies how the statistics of ∆*S_i_* time series changes upon increasing the scale from 100 events to a longer scale e.g., *i* = 10^3^ events.

## 3. Data and Analysis

The seismic data analysed in natural time come from the seismic catalog of the National Seismic Service (SSN) of the Universidad Nacional Autónoma de México (www.ssn.unam.mx) and cover the period 1 January 2012 until 20 October 2017. Since in this analysis *Q_k_* should be [11,15] proportional to the energy emitted during the *k*-th earthquake of magnitude *M_k_*, we assumed $Q_{k}\propto 10^{1.5M_{k}}$ [11]. To assure catalog completeness a magnitude threshold *M* ≥ 3.5 has been imposed. Furthermore, in order to investigate whether our results obey magnitude threshold invariance, we also repeat our calculations for *M* ≥ 4.0, as will be explained in the next Section.

Our calculations are made, as mentioned in the previous section, by means of a window of length *i* (= number of successive events) sliding each time by one event through the whole time series. The entropies *S* and *S_−_*, and therefrom their difference ∆*S_i_*, are calculated each time; we thus also form a new time series consisting of successive ∆*S_i_* values. The complexity measures λ*_i_* and Λ*_i_* are determined according to their definitions given in Equations (4) and (5), respectively.

## 4. Results

**Figure 1:**
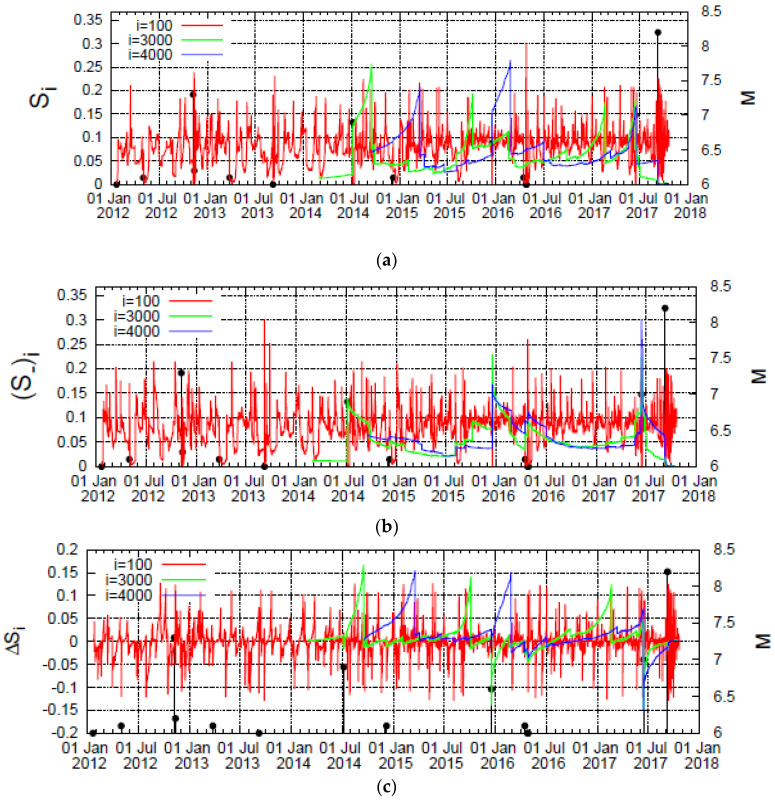
Plot of the entropies *S_i_* (**a**) and (*S_−_*)*_i_* (**b**) as well as the entropy change ∆*S_i_* under time reversal (**c**) versus the conventional time for the three scales *i* = 10^2^ (red), 3 × 10^3^ (green), and 4 × 10^3^ (blue) events when analysing all earthquakes with *M* ≥ 3.5. The vertical lines ending at circles depict the earthquake magnitudes, which are read in the right scale.

In Figure 1a–c we plot the values calculated for the quantities *S_i_*, (*S_−_*)*_i_*, and ∆*S_i_*, respectively, versus the conventional time for all *M* ≥ 3.5 earthquakes in the Chiapas region during the period 1 January 2012 to the date of occurrence of the M8.2 earthquake for the scales 10^2^, 3 × 10^3^, and 4 × 10^3^ events. The study of the first scale (10^2^ events) is needed for the calculation of the denominator of Equations (4) and (5), while the selection of the other scales (3 × 10^3^ events and longer) was made for the following reasons, as also explained in Ref. [7]. Since ∼11,500 earthquakes (*M* ≥ 3.5) occurred in this area from 1 January 2012 until the occurrence of the M8.2 earthquake on 7 September 2017, we find an average of around 170 earthquakes per month. We take into account that recent investigations by means of natural time analysis revealed that the fluctuations of the order parameter of seismicity exhibit [24] a minimum when a series of precursory low frequency (≤0.1 Hz) electric signals, termed Seismic Electric Signals (SES) activity (e.g., see [22]) (whose lead time is up to around 5.5 months [11]), is initiated. While this minimum of the order parameter of seismicity is observed during a period in which long-range correlations prevail between earthquake magnitudes, another stage appears before this minimum in which the temporal correlations between earthquake magnitudes exhibit a distinctly different behaviour, i.e., an evident anticorrelated behaviour [25]. The significant change between these two stages in the temporal correlations between earthquake magnitudes is likely to be captured by the time evolution of Δ*S_i_*, hence we started the presentation of our study of Δ*S_i_* in Ref. [7] from the scale of *i* ∼ 10^3^ events, i.e., around the maximum lead time of SES activities. This study led to the conclusion that at scales *i* = 3 × 10^3^ or longer (e.g., 4 × 10^3^ and 5 × 10^3^ events), a pronounced minimum becomes evident at the date 14 June 2017 (when a M7 earthquake occurred, i.e., almost 3 months before the M8.2 earthquake that struck Mexico’s Chiapas state). Interestingly, this minimum of Δ*S_i_* was found to exhibit magnitude threshold invariance. Hence, in the following we will present our results for these scales and in addition focus our attention on what happens before the occurrence of the M7 earthquake on 14 June 2017 and before the M8.2 earthquake on 7 September 2017. 

In particular, we will investigate the values of the complexity measures λ*_i_* and Λ*_i_* on the following 6 dates: On 1 June 2017 (i.e., almost two weeks before the occurrence of the M7 event on 14 June), on 14 June 2017 (upon the occurrence of the last event before the M7 earthquake on 14 June 2017), on 1 July 2017, on 1 August 2017, on 1 September 2017 (6 days before the M8.2 earthquake), and on 7 September 2017 (upon the occurrence of the last small event before the M8.2 earthquake).

### 4.1. Results for the Complexity Measure λ_i_

**Figure 2:**
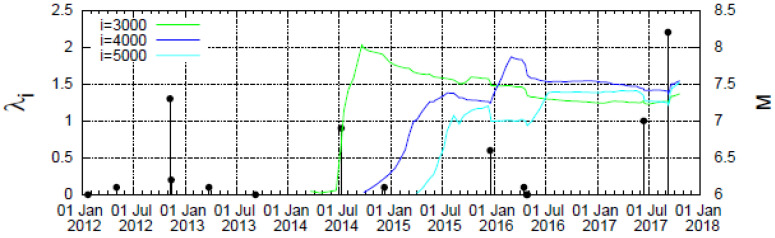
Plot of the values of the complexity measure *λ_i_* versus the conventional time that correspond to the scales *i* = 3 × 10^3^ (green), 4 × 10^3^ (blue), and 5 × 10^3^ (cyan) events when considering all earthquakes in the Chiapas region with *M* ≥ 3.5 since 2012.

**Figure 3:**
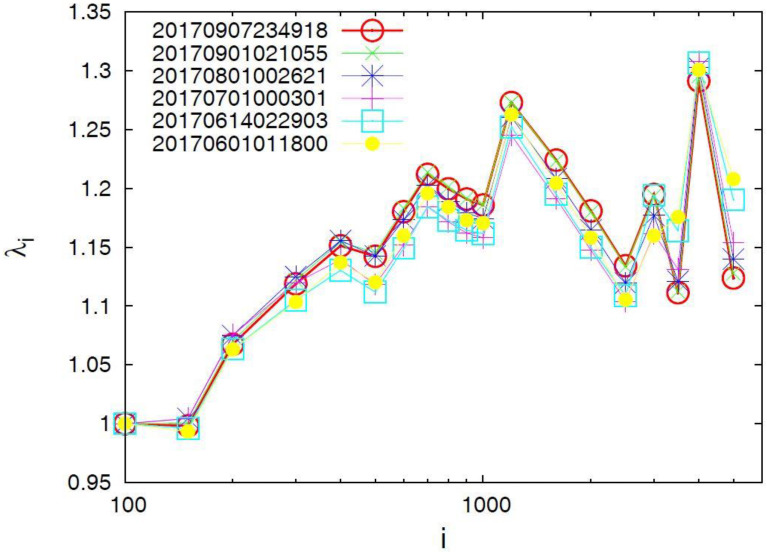
Plot of the complexity measure *λ_i_* versus the scale *i* (number of events) for all *M* ≥ 3.5 earthquakes in the Chiapas region since 1 January 2012. The *λ_i_* values are calculated for each *i* value at the following dates: 1 June 2017 (yellow solid circles), 14 June 2017 (cyan squares), 1 July 2017 (red plus), 1 August 2017 (blue star), 1 September 2017 (green cross), and 7 September 2017 (red circle, until the last event before the M8.2 earthquake on 7 September 2017).

We will now present our results for the complexity measure λ*_i_*, which is solely associated with the fluctuations *δS_i_* of the entropy in forward time. In Figure 2, we plot the λ*_i_* values versus the conventional time by considering all *M* ≥ 3.5 earthquakes from 1 January 2012 until the occurrence of the M8.2 earthquake. A close inspection of this figure in all scales investigated does not reveal any remarkable change before the occurrence of the M8.2 earthquake. The same conclusion is drawn when we plot in Figure 3 the λ*_i_* values versus the scale *i* of the number of *M* ≥ 3.5 events that occurred in the Chiapas region from 1 January 2012 until the dates mentioned above before the two earthquakes, i.e., the M7 earthquake on 14 June 2017 and the M8.2 earthquake on 7 September 2017. Remarkably, the resulting values for all six dates coincide for each *i* value without showing any precursory variation. In other words, even when considering the λ*_i_* value on 7 September 2017, upon the occurrence of a small event just before the M8.2 earthquake, the value at *i* = 5000 does not significantly differ from the other λ*_i_* values that correspond, for example, to that of 1 June 2017, i.e., more than 3 months before the M8.2 earthquake occurrence. 

### 4.2. Results for the Complexity Measure Λ_i_

**Figure 4:**
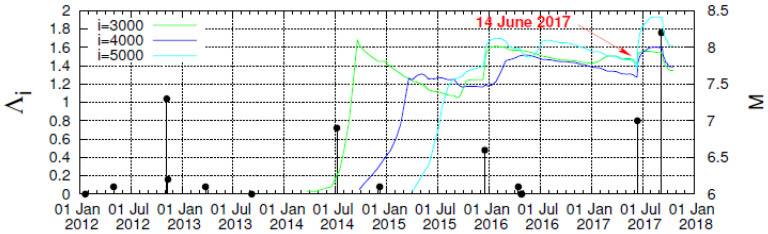
Plot of the values of the complexity measure Λ*_i_* versus the conventional time that correspond to the scales *i =* 3 × 10^3^ (green), 4 × 10^3^ (blue), and 5 × 10^3^ (cyan) events when considering all earthquakes in the Chiapas region with *M* ≥ 3.5 since 2012.

In Figure 4 we plot the Λ*_i_* values versus the conventional time by considering all *M* ≥ 3.5 earthquakes in the Chiapas region from 1 January 2012 until the occurrence of the M8.2 earthquake. An inspection of this figure reveals that upon the occurrence of the M7 earthquake on 14 June 2017 an abrupt increase of Λ*_i_* is observed in all three scales.

**Figure 5:**
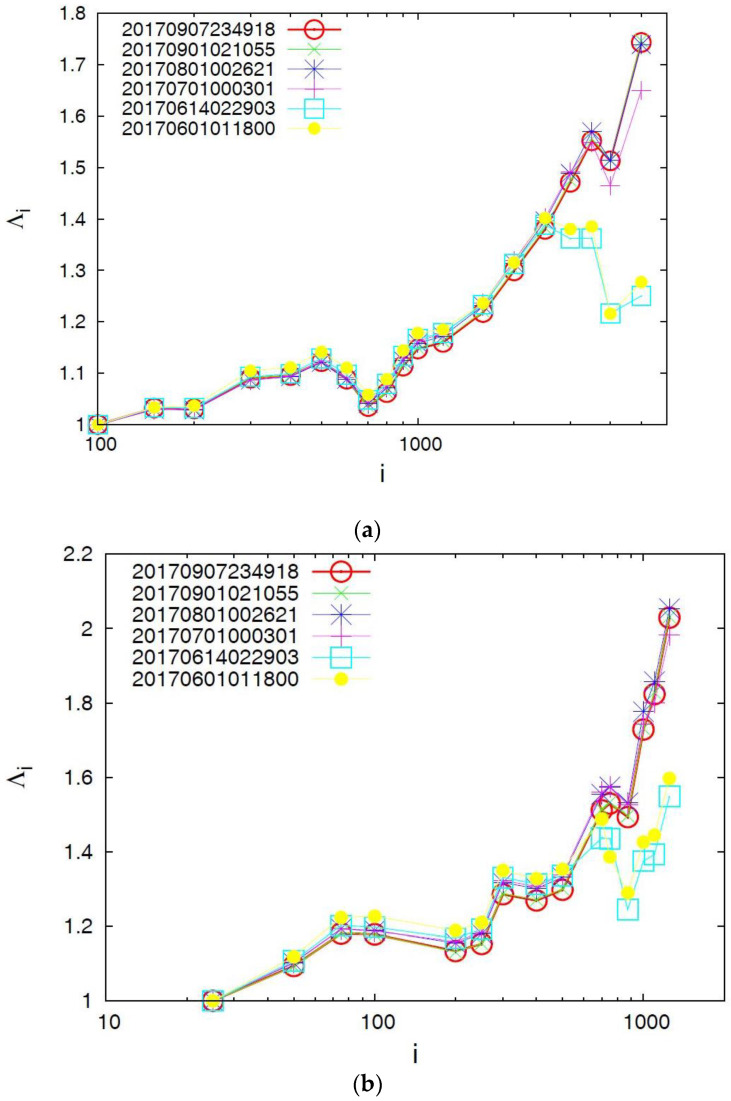
Plot of the complexity measure Λ*_i_* versus the scale *i* (number of events) for all *M* ≥ 3.5 earthquakes (**a**) as well as for all *M* ≥ 4.0 earthquakes (**b**) in the Chiapas region since 1 January 2012. The Λ*_i_* values are calculated for each *i* value at the following dates: 1 June 2017 (yellow solid circles), 14 June 2017 (cyan squares), 1 July 2017 (red plus), 1 August 2017 (blue star), 1 September 2017 (green cross) and 7 September 2017 (red circle, until the last event before the M8.2 earthquake on 7 September 2017).

Furthermore, in Figure 5 we plot the Λ*_i_* values versus the scale *i* of the number of *M* ≥ 3.5 events in the Chiapas region from 1 January 2012 until the six dates mentioned above before the two earthquakes, i.e., the M7 earthquake on 14 June 2017 and the M8.2 earthquake on 7 September 2017. We observe that for the scales *i =* 3000 events and larger an evident abrupt increase of the Λ*_i_* value is observed upon the occurrence of the M7 earthquake on 14 June 2017. This date of the abrupt increase of Λ*_i_* remains invariant upon changing the magnitude threshold, for example, see the result also plotted in Figure 5 by increasing the magnitude threshold to *M ≥* 4.0 instead of *M* ≥ 3.5 and considering that the number of earthquakes then decreases by a factor of around 4.

## 5. Discussion

While the complexity measure Λ*_i_* does exhibit an evident precursory change almost three months before the M8.2 earthquake, the complexity measure *λ_i_* does not. This should not be considered as surprising in view of the following: Λ*_i_* is associated with the fluctuations of the entropy change under time reversal, and we know that ∆*S_i_* is a key quantity to determine the time of an impending dynamic phase transition [20] as, for example, is the case in sudden cardiac death risk. This is also the case with earthquakes because the observed earthquake scaling laws (e.g., [26]) indicate the existence of phenomena closely associated with the proximity of the system to a critical point. Thus, taking the view that a strong earthquake is a critical phenomenon (dynamic phase transition), it may not come as a surprise that the fluctuations of ∆*S_i_*, and hence Λ*_i_*, may serve for the estimation of the time of its occurrence. We note that in [12] the predictability of the OFC model was studied by using the entropy change under time reversal. It was found that the value of Δ*S_i_* exhibits a clear minimum (if we define Δ*S* = *S* − *S*_−_) [7] or maximum (if we define Δ*S* = *S*_−_ − *S*) [12] before large avalanches in the OFC model, thus this minimum provides a decision variable for the prediction of a large avalanche. The prediction quality of this minimum was studied, following [27], by choosing the receiver operating characteristics (ROC) graph [28], see Figure 2 of [12]. The ROC graph was constructed for the OFC model, and it was found that the predictions made on the basis of Δ*S* are statistically significant, and, as concerns the origin of their predictive power, this should be attributed to the fact that Δ*S* is able to catch the ‘true’ time arrow.

**Figure 6:**
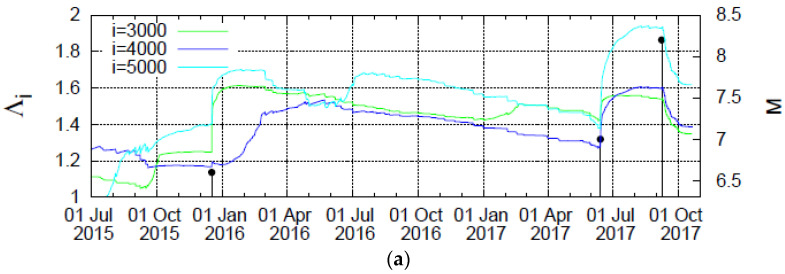

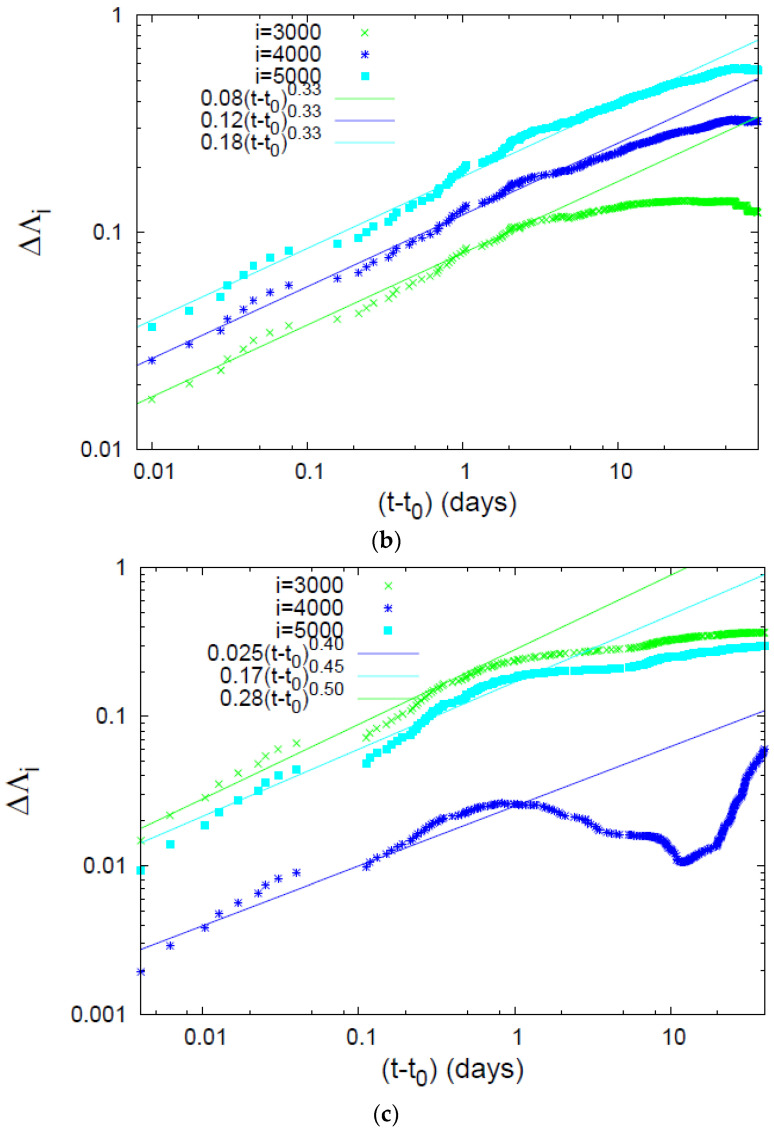
(**a**) The same as in Figure 4 but in an expanded timescale; (**b**,**c**) plot of the change ΔΛ*_i_* of the complexity measure Λ*_i_* after the occurrence of the M7.0 earthquake on 14 June 2017 (**b**) and the M6.6 earthquake on 17 December 2015 (**c**) versus the time elapsed (*t* − *t*_0_) in days. In both cases, the value of t_0_ has been selected approximately 30 min after earthquake occurrence.

To further study the increase of Λ*_i_* possibly associated with strong earthquakes, we now investigate the following: At first glance, one may claim that the behavior of Λ*_i_* observed on 14 June 2017 seems to be very similar to what happens around the end of 2015, and both changes seem to be related to earthquakes around these times. In order to examine whether such a similarity is true, we now depict in Figure 6a the complexity measures Λ*_i_* versus the conventional time in an expanded time scale for *M* ≥ 3.5. A close inspection of this figure reveals that all the three complexity measures Λ_3000_, Λ_4000,_ and Λ_5000_ (which stand for the Λ*_i_* values at the scales *i =* 3000, 4000, and 5000, respectively) exhibit a strong and abrupt increase on 14 June 2017 (after the occurrence of a M7.0 earthquake). Such a strong and abrupt increase for *all* these three complexity measures is *not* observed at any other time. In particular, on 17 December 2015 after the occurrence of a M6.6 earthquake, only Λ_3000_ and Λ_5000_ exhibit a strong and abrupt increase, while the corresponding increase for Λ_4000_ is much smaller and obviously does not scale with *i*. The behavior of the increase ΔΛ*_i_* (≡Λ*_i_*(*t*) − Λ*_i_*(*t_EQ_*)) of the complexity measure Λ*_i_* upon the occurrence of a strong earthquake at *t_EQ_* is further studied in Figure 6b,c for the earthquakes of 14 June 2017 and 17 December 2015, respectively. An inspection of these two figures shows that while the abrupt increase on 14 June 2017 exhibits a scaling behavior of the form

(6) $$\Delta \Lambda_{i}=A\;{\left(t-t_{0}\right)}^{c},$$

where the pre-factors *A* are proportional to *i* and the exponent *c* is independent of *i* with a value close to 0.33, this does not hold for the case of 17 December 2015. We also comment that quite interestingly there exists a striking similarity between the results shown in Figure 6b and the seminal work by Lifshitz and Slyozov [29], and independently by Wagner [30], who found a form like that of Equation (6) with an exponent close to 1/3 for the time growth of the characteristic size of the minority phase droplets in phase transitions (e.g., [31]).

**Figure 7:**
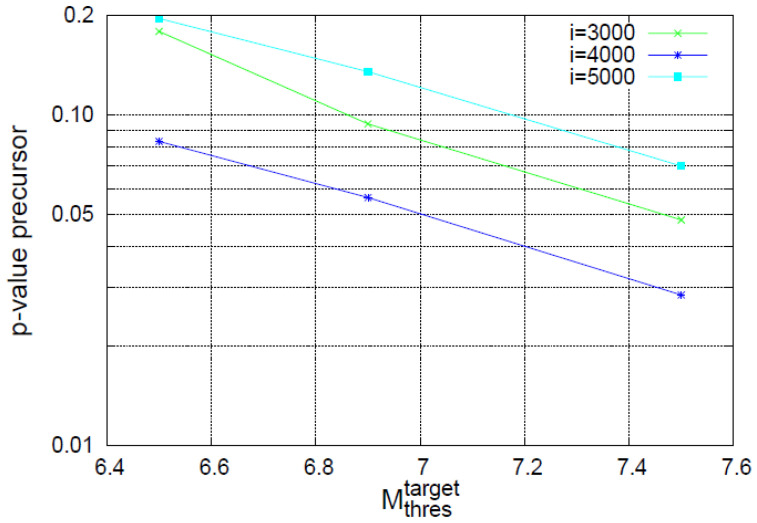
Results from the event coincidence analysis between the earthquake events of magnitude $M>M_{thres}^{target}$ and the events of abrupt increase of Λ*_i_* (see Figure 4 and Figure 6a). The probability to obtain by chance (*p*-value) the observed results when considering the events of abrupt increase of Λ*_i_* as precursors to strong earthquakes versus the threshold $M_{thres}^{target}$ for various scales *i*. The *p*-values have been obtained by using the compute code eca.es of the CoinCalc package [32] of R.

Concerning the statistical significance of the abrupt increase of the complexity measure Λ*_i_* three months before the M8.2 Chiapas earthquake as a precursor to strong earthquakes, we employ the most recent method of event coincidence analysis [32] as implemented by the CoinCalc package in R. More specifically, for every month we estimate the largest earthquake magnitude *M*_max_ observed and compare it with a threshold $M_{thres}^{target}$, if $M_{\max}>M_{thres}^{target}$ a strong earthquake event occurred in that month. The precursor event time series is composed by the abrupt increase in events of Λ*_i_* (see Figure 4 and Figure 7a) and exhibits two events for *i =* 5000 and 3000 (one in December 2015 and one in June 2017), while for *i =* 4000 a single event (in June 2017). Figure 7 shows how the corresponding *p*-values vary upon increasing the threshold $M_{thres}^{target}$ to obtain the real situation by chance. We observe that only in three out of the nine results shown, these *p*-values exceed 10%, while for the largest threshold $M_{thres}^{target}$ the corresponding *p*-values are 4.8%, 2.8%, and 7.0% for *i =* 3000, 4000, and 5000, respectively, pointing to the statistical significance of the precursor under discussion.

Similar results to those obtained here have been found for other time series prior to large earthquakes in other places. As a characteristic example we mention the M9 Tohoku mega-earthquake that occurred in Japan on 11 March 2011. In particular, the complexity measure Λ*_i_*_,_ associated with the fluctuations of the entropy change of the seismicity in Japan (with *M* ≥ 3.5), was studied during the period from 2000 to 11 March 2011 and found to exhibit an abrupt increase upon the occurrence of a M7.8 earthquake on 22 December 2010. Remarkably, on the same date [25] the temporal correlations between earthquake magnitudes exhibited anticorrelated behavior with the lowest value (≈0.35) ever observed of the exponent in the detrended fluctuation analysis [33,34,35] we employed in that study. Details on the interconnection between the changes in the temporal correlations between earthquake magnitudes and the precursory Δ*S* minimum in Japan will be published shortly elsewhere.

**Figure 8:**
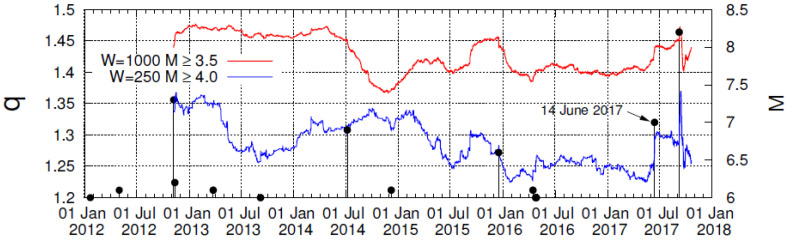
The entropic index *q* versus conventional time for sliding windows *W* = 1000 (red) and *W* = 250 (blue) consecutive earthquakes in the Chiapas region since 2012 for *M* ≥ 3.5 and *M* ≥ 4.0, respectively.

Finally, we comment on the results obtained on the basis of the pioneering work of Tsallis [36], which introduced a non-additive entropy *S_q_* (e.g., see [37,38]). This has found application in the physics of earthquakes and especially in the description of the asperities [39,40] in the faults on which earthquakes occur through the entropic index *q*. Based on the earthquake magnitude distribution one can obtain [41,42] entropic index *q* and study its variation with time as we approach a strong EQ (for a recent review see [43]). Figure 8 depicts the *q*-value versus conventional time as it is estimated [42] for sliding windows of *W* = 1000 and *W* = 250 consecutive earthquakes in the Chiapas region for *M* ≥ 3.5 and *M* ≥ 4.0, shown as red and the blue lines, respectively. We observe that before the occurrence of the M8.2 Chiapas earthquake, the *q*-value starts to grow gradually during May 2017 and exhibits an abrupt increase upon the occurrence of the earthquake on 14 June 2017 for both magnitude thresholds. This behavior is reminiscent to that observed before the 1995 Kobe earthquake in Japan (e.g., see Figure 3 of [44]).

## 6. Conclusions

It was found that the complexity measure λ*_i_* studied during the period 2012–2017, which is solely associated with the fluctuations of entropy in forward time, does not exhibit any obvious precursory change before the M8.2 earthquake on 7 September 2017.

On the other hand, the complexity measure Λ*_i_* associated with the fluctuations of entropy change under time reversal shows an evident increase on 14 June 2017, accompanied by an abrupt increase of the Tsallis entropic index *q*. Interestingly, on the same date reported by Sarlis et al. in [7], the entropy change under time reversal has been found to exhibit a minimum, as was found in the OFC model [12].
